# Supplementary material for: Are carbon emissions trading and green financial instruments synergistic? -Comprehensive quantitative research based on content analysis
Source: PLoS One. 2024 Mar 7;19(3):e0298601. doi: 10.1371/journal.pone.0298601 (PMC10919601; doi:10.1371/journal.pone.0298601)
Supplement: S1 Data — (ZIP) [file pone.0298601.s001.zip › Data/地级市Z_F工作报告.zip!/▒▒╛⌐/└┤╫╘╣1⁄2╓┌║┼í╛┬φ┐╦╩2╛▌═°í┐_2018.docx]

——2018年1月24日在北京市第十五届人民代表大会第一次会议上

北京市代市长　陈吉宁

各位代表：

　　现在，我代表北京市人民政府，向大会报告过去五年政府工作，对今年工作提出建议，请予审议，并请市政协各位委员提出意见。

　　一、过去五年工作回顾

　　过去五年，是北京发展历史上极不平凡的五年。习近平总书记两次视察北京并发表重要讲话，明确了北京全国政治中心、文化中心、国际交往中心、科技创新中心的城市战略定位，为我们“建设一个什么样的首都，怎样建设首都”统一了思想、深化了认识、指明了方向。去年6月，习近平总书记主持中央政治局常委会会议，亲自听取北京城市总体规划编制工作汇报；9月，党中央、国务院正式批复《北京城市总体规划(2016年-2035年)》，为我们建设好伟大社会主义祖国的首都、迈向中华民族伟大复兴的大国首都、国际一流的和谐宜居之都绘就了宏伟蓝图，提供了基本依据。

　　五年来，在以习近平同志为核心的党中央坚强领导下，在中共北京市委直接领导下，在市人大及其常委会监督支持下，我们以习近平新时代中国特色社会主义思想为指引，坚持稳中求进工作总基调，牢固树立新发展理念，深入落实首都城市战略定位，大力推进京津冀协同发展，加快疏功能、转方式、治环境、补短板、促协同、惠民生，推动首都现代化建设取得重大进展。

　　(一)城市发展深刻转型。立足首都城市战略定位，深刻把握“都”与“城”、“舍”与“得”、疏解与提升、“一核”与“两翼”的关系，推动城市发展理念和发展方式加快转变。紧紧抓住疏解非首都功能这个“牛鼻子”，有力开展疏解整治促提升专项行动，制定实施严格的新增产业禁止和限制目录，累计不予办理登记业务1.86万件，关停退出一般制造业企业1992家，调整疏解各类区域性专业市场594家，大力整治违法建设、“开墙打洞”、占道经营、背街小巷环境脏乱等突出问题，解决了一批多年想解决而没有解决的难题，城市面貌发生积极变化。推动教育、医疗等公共服务资源均衡布局，有序引导功能疏解。加快城市副中心规划建设，精心编制副中心控制性详细规划，行政办公区等各项重点工程建设进展顺利。

　　(二)京津冀协同发展取得阶段性成效。全力支持雄安新区规划建设，与河北省签订战略合作协议，确定了8个重点合作领域及一批先期支持项目。全面落实《京津冀协同发展规划纲要》，交通、生态环保、产业三个重点领域实现率先突破。国家高速公路网7条首都放射线北京段全部建成，新机场航站楼封顶封围。实现区域空气重污染联防联控，空气质量明显改善。新增京冀生态水源保护林50万亩，永定河等流域生态保护统一规划、协同治理工作取得积极进展。加强产业转移承接重点平台建设，推进京津冀全面创新改革试验，2015年以来与津冀的技术合同成交额近470亿元。开展异地就医住院医疗费用直接结算，实现社会保险互联互认，推动公共服务合作共享。

　　(三)科技创新能力和经济发展质量稳步提升。积极适应和引领经济发展新常态，深入推进供给侧结构性改革，着力构建高精尖经济结构，首都经济在转型发展中稳中有进、稳中向好。初步统计，地区生产总值年均增长7.1%、2017年达到2.8万亿元，一般公共预算收入年均增长10.4%、达到5430.8亿元，第三产业比重达到80.6%；万元地区生产总值能耗、水耗和二氧化碳排放分别累计下降22.5%、22%和28.2%，能源利用效率位居全国首位。落实全国科技创新中心建设总体方案，加速“三城一区”主平台建设，发挥中关村国家自主创新示范区引领带动作用，出台一系列先行先试政策，涌现一批重大标志性原创性成果和创新型企业，示范区规模以上企业总收入超过5万亿元、是2012年的两倍。实施《中国制造2025》北京行动纲要，发布新一代信息技术等10个高精尖产业发展指导意见及财政、土地、人才等一揽子支持政策，形成了全市上下推动高精尖产业发展的良好氛围。

　　(四)改革开放取得新突破。坚决落实中央全面深化改革决策部署，以开放促改革，以改革促发展。“放管服”改革扎实推进，精简行政审批事项1065项、精简比例达到68%，全市清理各类证明206项；“先照后证”等商事制度改革落地见效，优化营商环境26条措施启动实施。经济领域改革多点突破，“营改增”试点改革全面推开，政府性收支全部纳入预算管理，政府和社会资本合作模式有效推广，阶梯水价、电价、气价和公共交通票制票价改革平稳实施，市属一级企业公司制改革全部完成，农村集体经营性建设用地入市试点稳步推进。深化城市管理和执法体制改革，组建市区两级新的城市管理机构，城管执法力量下沉到街乡镇。调整组建市卫生计生委、规划国土委，完善市、区、街乡镇三级食品药品监管体系。大力实施医药分开综合改革，3700多家医疗机构取消药品加成，实行药品阳光采购，开展首批医疗服务价格规范调整，解决以药养医问题取得重要突破。

　　率先开展服务业扩大开放综合试点，开放型经济达到新水平。2017年实际利用外资243.3亿美元、跃居全国第一，其中服务业利用外资超九成。促进对外贸易转型升级，服务贸易额约占全国的1/5。积极对接服务“一带一路”建设，亚洲基础设施投资银行、丝路基金落户北京。

　　(五)“大城市病”治理力度显著加大。实施清洁空气行动计划，发挥环保督察“利剑”作用，层层压实责任，年燃煤总量从2270万吨减至600万吨以内，淘汰老旧机动车216.7万辆，清理整治“散乱污”企业1.1万家，2017年细颗粒物年均浓度为58微克/立方米，圆满完成第一阶段国家大气污染治理任务。持续实施缓解交通拥堵专项行动计划，新增轨道交通166公里、总里程达到608公里，公交专用道总里程达到907公里，完成城六区次支路建设114条、堵点乱点治理240个，规范发展共享单车，中心城绿色出行比例达到72%。全面推行河长制，连续实施两个污水处理三年行动方案，污水处理率提高到92%，再生水年利用量达到10.5亿立方米。坚持节水优先，南水北调水安全高效利用，密云水库蓄水量超过20亿立方米。实施垃圾处理设施建设三年行动计划，生活垃圾日处理能力达到2.4万吨，资源化率达到57%。开展农村人居环境综合整治，累计创建美丽乡村1300个。城市生态空间大幅度增加，超额完成平原百万亩造林任务，新增城市绿地4000公顷，全市森林覆盖率由2012年的38.6%提高到43%。

　　(六)人民生活持续改善。贯彻以人民为中心的发展思想，累计办理重要民生实事146件。城镇登记失业率保持在1.5%左右的较低水平。全市居民人均可支配收入增长与经济增长同步，2017年达到5.7万元、同比实际增长6.9%，低收入农户收入增速连续7年快于全市农民。整合城乡居民基本医疗保险制度，统一城乡低保标准，城乡一体的社会保障体系率先形成。实施两期学前教育三年行动计划，累计增加学位17万个。深化考试招生制度改革，义务教育免试就近入学比例不断提高。医疗服务体系进一步完善，预约挂号、分时段就诊等创新服务全面推开，群众就医更加方便。落实居家养老服务条例，建设“三边四级”养老服务体系，建成街乡镇养老照料中心208个、社区养老服务驿站380家。实施提高生活性服务业品质行动计划，“一刻钟社区服务圈”城市社区覆盖率达到87.5%。公共文化服务体系更加完善，文艺精品不断涌现，文化创意产业活力增强，群众体育蓬勃发展。加大住房保障力度，累计建设筹集各类保障性住房49万套、竣工42万套，完成17.5万户棚户区改造和5000万平方米老旧小区综合整治任务，改善了群众居住条件。加强房地产市场调控，发布2017-2021年住宅用地供应计划，探索建立共有产权住房制度。截至2017年底，新建商品房价格连续15个月环比不增长，二手房价格指数连续8个月回落，房地产市场总体平稳。扎实做好对口支援合作工作，实施各类援助项目2650项，有力推动受援地区脱贫攻坚。

　　社会保持和谐稳定。加强枢纽型社会组织建设，基本实现社会服务管理网格全覆盖。大力支持国防和军队改革，积极配合驻京部队全面停止有偿服务，深化国防动员和双拥共建，人民防空工作得到加强，军民融合发展成效明显。妇女儿童、残疾人事业取得新发展，民族、宗教、对台、侨务工作进一步加强。以“四个最严”要求强化食品药品安全监管，持续加大安全生产工作力度，严守安全底线。防汛减灾工作扎实有效，城市排涝能力明显增强。涉法涉诉信访工作改革稳步推进，深入开展矛盾纠纷排查化解。广泛动员群众参与平安北京建设，形成一批群防群治品牌。深入落实“六住”措施，反恐防恐能力不断提升。积极推进社会治安防控体系转型升级，深化基层警务改革创新，依法严厉打击各类违法犯罪行为，保持了首都和谐稳定的良好局面。

　　各位代表！五年来，在党中央、国务院的亲切关怀下，在各有关方面的大力支持下，北京携手张家口成功申办2022年冬奥会，各项筹办工作正在扎实有序推进。我们自觉服务党和国家工作大局，坚持首善标准，切实履行首都职责，圆满完成亚太经合组织领导人非正式会议、中国人民抗日战争暨世界反法西斯战争胜利70周年纪念活动、“一带一路”国际合作高峰论坛等重大服务保障任务，全力以赴做好党的十九大服务保障和维稳安保工作，向党和人民交上了一份合格答卷。

　　五年的成就是全方位的，五年的变革是深层次的，北京发生了令人鼓舞的变化，发展质量提高了，生态环境改善了，城乡面貌亮丽了，群众获得感增强了。这些成绩的取得，最重要、最根本的是有以习近平同志为核心的党中央坚强领导，有习近平新时代中国特色社会主义思想的科学指引。这也是全市人民在中共北京市委的领导下齐心协力、攻坚克难、努力奋斗的结果。成绩来之不易，我们要倍加珍惜。在此，我谨代表北京市人民政府，向全市人民，向全体人大代表、政协委员，向各民主党派、各人民团体和各界人士，向中央和国家机关各部门各单位、各兄弟省区市，向驻京解放军和武警部队官兵，向所有关心支持首都建设的香港特别行政区同胞、澳门特别行政区同胞、台湾同胞、海外侨胞和国际友人，表示衷心感谢！

　　我们清醒地认识到，我国社会主要矛盾已经发生变化，与首都承担的职责使命相比，与城市总体规划提出的目标要求相比，与人民对美好生活的向往相比，发展还面临许多挑战，工作也存在诸多不足：人口资源环境矛盾依然十分突出，城市精细化、专业化管理亟须加强，“大城市病”治理任重道远；全国科技创新中心的引擎作用不够，对高质量发展的支撑带动有待增强；城乡、区域发展不协调，公共服务和便民设施尚有诸多短板；开放发展活力不足，国际化服务环境和能力还有明显差距；城市公共安全形势不容乐观，“11·18”重大事故教训深刻、令人痛心；群众办事难问题仍较突出，政府工作能力和作风需要大力提升。对此，我们一定要保持清醒头脑，勇于直面问题，以对人民高度负责的担当精神，在今后工作中切实加以解决。

　　二、今后五年工作思路和2018年重点任务

　　今后五年是实现“两个一百年”奋斗目标的历史交汇期。党的十九大作出了中国特色社会主义进入新时代的重大政治判断，描绘了决胜全面建成小康社会、开启全面建设社会主义现代化国家新征程、实现中华民族伟大复兴中国梦的宏伟蓝图，吹响了新时代沿着中国特色社会主义道路奋勇前进的嘹亮号角。我们要以高度的政治责任感和历史使命感，全面贯彻党的十九大精神，认真落实市第十二次党代会的决策部署，推动习近平新时代中国特色社会主义思想在京华大地落地生根，进一步形成生动实践。

　　在今后工作中，要着重把握好以下要求：必须牢固树立政治意识、大局意识、核心意识、看齐意识，把维护党中央权威和集中统一领导作为最高政治原则和根本政治规矩来执行；必须坚持稳中求进工作总基调，处理好“稳”和“进”的辩证统一关系，把握好工作节奏和力度；必须深入落实首都城市战略定位，着力加强“四个中心”功能建设，提高“四个服务”水平；必须推动高质量发展，牢固树立新发展理念，强化创新驱动，加快构建高精尖经济结构，着力破解“大城市病”，把高质量要求体现在城市发展的各个方面；必须大力推动改革开放，激发市场活力和创造力，提高对外开放服务水平；必须坚持以人民为中心，发挥人民主体作用，积极回应群众关切，努力解决广大市民关心的突出问题，增强群众获得感幸福感安全感。

　　进入新时代，迈向新征程，我们要更加自觉肩负起首都的职责使命，抓好“三件大事”，打赢“三大攻坚战”。“三件大事”，就是要精心组织实施新一版北京城市总体规划；以疏解北京非首都功能为“牛鼻子”推动京津冀协同发展；全力筹办好2022年北京冬奥会、冬残奥会。“三大攻坚战”，就是要打好防范化解重大风险攻坚战，重点是防控金融风险，切实加强政府债务管理，履行地方金融监管职责，打击非法集资等违法违规金融活动，坚决守住不发生系统性金融风险的底线；打好精准脱贫攻坚战，不仅要促进本市低收入农户增收，更要坚决落实好中央部署的扶贫帮扶任务，助力受援地区如期脱贫；打好污染防治攻坚战，重点是打赢蓝天保卫战、碧水攻坚战、突出问题歼灭战，特别是大气污染防治，随着第一阶段治理任务完成，污染源结构发生较大变化，持续改善空气质量的任务异常繁重，要充分估计其长期性、艰巨性和复杂性，既打好攻坚战，又打好持久战。

　　2018年是贯彻党的十九大精神的开局之年，是改革开放40周年，是决胜全面建成小康社会、实施“十三五”规划承上启下的关键一年。今年政府工作的总体要求是：全面深入学习贯彻党的十九大精神，以习近平新时代中国特色社会主义思想为指引，坚持稳中求进工作总基调，坚持新发展理念，紧扣我国社会主要矛盾变化，按照高质量发展的要求，统筹推进“五位一体”总体布局和协调推进“四个全面”战略布局，坚持以供给侧结构性改革为主线，统筹推进疏功能、稳增长、促改革、调结构、惠民生、防风险各项工作，着力加强“四个中心”功能建设，提高“四个服务”水平，抓好“三件大事”，打好“三大攻坚战”，更加奋发有为地推动首都新发展。

　　今年全市经济社会发展主要预期目标是：地区生产总值增长6.5%左右；一般公共预算收入增长6.5%左右；居民消费价格涨幅控制在3%以内；城镇调查失业率、城镇登记失业率分别控制在5%以内和3%以内；全市居民人均可支配收入增长与经济增长同步；万元地区生产总值能耗、二氧化碳排放分别下降2.5%和3%左右，万元地区生产总值水耗下降3%左右，细颗粒物年均浓度力争继续下降。

　　今年要着力做好以下重点工作。

　　(一)全面实施城市总体规划，优化提升“四个中心”功能。

　　深入贯彻落实党中央、国务院批复精神，坚持一张蓝图干到底，以钉钉子精神抓好规划组织实施，切实发挥规划的战略引领和刚性管控作用，努力把首都规划好、建设好、管理好。

　　创新规划实施体制机制。实行全域规划总量管控，划定城市开发边界和生态控制线，严控建设用地规模，实现生态用地动态增长。建立各区城乡建设用地增减挂钩机制，严格落实拆建比、拆占比，全市城乡建设用地年度减量30平方公里以上，适度提高居住用地及其配套用地比重，推动职住平衡。建立市区规划分级管理体制，整合配强区级规划国土部门力量，市级层面负责总体管控和制定政策导则标准，区级层面负责组织区域控规编制和规划任务落地。以立项和开工为节点，并联办理审批事项，压缩时限30%左右。以第三方评估方式实施年度城市体检，加强规划实施的监督考核问责，坚决维护总体规划的严肃性权威性。

　　抓好规划实施重点任务落地。按照“一核一主一副、两轴多点一区”城市空间布局，编制分区规划实施要点和区域控制性详细规划，深入开展城市设计导则、标准和政策规范研究制定工作，实现多规合一、全域管控。同步开展物流、公共服务、地下空间和人防设施、市政基础设施、绿地系统等专项规划，增强城市发展的整体性、协调性，保证城市安全高效运行。编制核心区控规，抓好长安街及其延长线规划管控和综合整治，制定中轴线申遗综合整治规划实施计划；优化中心城区空间布局，编制统筹腾退空间再利用规划，提升首都功能服务保障能力。高质量完成副中心各项规划，抓紧编制顺义、大兴、亦庄、昌平、房山等新城控制性详细规划，完善基础设施和公共服务设施，提高综合承载能力，强化多点支撑作用。编制浅山区保护规划，加强违法占地、违法建设专项治理，将浅山区建设成为首都生态文明示范区。

　　(二)坚定有序推进疏解整治促提升专项行动，提升城市品质、改善人居环境。

　　紧紧抓住疏解非首都功能这个“牛鼻子”，坚持疏解整治与优化提升同步推进，努力营造整洁有序的城市面貌和便利宜居的生活环境。

　　持续抓好疏解整治工作。修订细化新增产业禁止和限制目录，退出一般制造业企业500家，疏解退出城六区二级及以下市属国有企业40家以上，继续疏解区域性专业市场。以医院、学校等公共服务资源空间布局调整为抓手，加强薄弱地区公共服务能力建设，引导中心城区功能和人口有序转移，保持合理的职业结构。启动北京联合大学新校区规划选址，抓紧北京工商大学、北京电影学院和北京信息科技大学等新校区建设；实现天坛医院新院区运行及老院区腾退，扎实推进北京口腔医院整体迁建和友谊医院顺义院区、同仁医院亦庄院区二期等项目建设。拆除违法建设4000万平方米以上，确保新生违法建设零增长。启动新一轮老旧小区综合整治，继续开展“开墙打洞”治理、架空线入地、广告牌匾规范设置等专项工作，完成核心区615条街巷环境整治任务。全力推进“一绿”地区城市化，试点探索“二绿”地区城乡融合发展新路径，完成100个市级挂账重点地区综合治理任务，让绿色成为城乡结合部的主色调。

　　更加注重优化提升。制定城市有机更新办法，实施街区设计导则，出台公共空间改造提升三年行动方案，推进城市修补和生态修复。建立全市拆违腾退土地“留白增绿”台账，实施拆后土地还绿1600公顷，新增10处城市休闲公园和100公里健康绿道。持续提高生活性服务业品质，建设提升基本便民商业网点1400个，其中蔬菜零售网点500个。推进“厕所革命”，完成580座各类公共厕所改造升级。制定回龙观、天通苑地区公共服务和基础设施优化提升三年行动计划，实施回龙观至上地自行车专用道、天通苑综合文化中心等13个项目，着力解决大型居住区群众关心的短板问题。

　　(三)深入推进京津冀协同发展，提高城乡区域协调发展水平。

　　从今年起，京津冀协同发展将向中期目标迈进，冬奥会、冬残奥会进入北京周期，要抓住新的历史机遇，充分发挥北京“一核”作用，推动雄安新区与城市副中心两翼联动，带动城乡统筹和一体化发展。

　　纵深推进重点领域合作。制定实施本市京津冀协同发展新三年行动计划。全方位支持雄安新区建设，落实与河北省签订的战略合作协议，加紧学校、医院等项目落地，推进雄安新区中关村科技园建设，切实做到雄安新区需要什么就支持什么。扎实推进交通一体化，全面消除市域内国家高速公路“断头路”，推进京张高铁、延崇高速等项目建设，实现兴延高速、首都地区环线通州至大兴段全线贯通。深入开展区域污染联防联控和生态环境保护，推进永定河综合治理与生态修复，加快京津风沙源治理二期等生态工程建设。加强产业对接协作，巩固提升“4+N”产业合作格局。全力抓好新机场这一重大标志性工程，基本完成主体工程建设，启动临空经济区起步区建设。加强教育、医疗、社保等基本公共服务合作，推进环首都1小时鲜活农产品流通圈建设。

　　加大城市副中心建设力度。落实副中心控制性详细规划，加快建设一批基础设施、公共服务、生态环境等重点工程，全面增强副中心综合承载力和吸引力。推进城市绿心建设，以生态的办法治理水系、恢复湿地，努力建设水城共融的生态城市、蓝绿交织的森林城市。抓紧建设人大附中通州校区、安贞医院通州院区和剧院、图书馆、博物馆等重大项目，进一步研究制定高标准配置优质公共服务资源方案。实施广渠路东延、宋梁路北延等项目，加强各类配套服务设施建设。围绕行政办公、商务服务、文化旅游三大主导功能，发展新产业新业态，推进运河商务区、环球主题公园等建设，促进产城融合。创新城市综合管理体制，在新型城镇化、投融资体制机制等方面迈出更大改革步伐。平稳有序做好市级机关和市属行政部门搬迁工作，推进行政办公区二期工程建设。按照统一规划、统一政策、统一管控的原则，处理好通州区与北三县关系，完善统筹协调机制。

　　实施乡村振兴战略，促进城乡融合发展。要把城乡统筹、区域协调放到京津冀协同发展大格局中谋划，在确保生态安全的前提下，提升城乡协调发展、绿色发展的质量和水平。按照产业兴旺、生态宜居、乡风文明、治理有效、生活富裕的总要求，编制乡村振兴战略规划，落实美丽乡村建设三年专项行动计划，启动1000个村庄人居环境整治。支持市属国有企业参与乡村振兴规划建设。以“三块地”为重点深化农村改革，完善承包地“三权”分置制度，统筹推进集体经营性建设用地入市和征地制度改革，持续推进乡镇统筹利用集体产业用地试点，规范引导闲置农宅盘活利用。推进农村集体产权制度和集体经济组织制度改革，深化集体林权制度和国有林场改革。突出农业生态功能，加强农业科技创新，继续推进农业“调转节”，积极打造农产品绿色优质安全示范区。新增农村劳动力转移就业4万人以上，健全生态林管护员岗位补贴动态增长机制，启动实施新一轮山区农民搬迁，对全市低收入农户高效实施“六个一批”精准帮扶，确保农民持续增收。制定实施新的城市南部地区发展三年行动计划，优先解决生态环境、公共服务、交通设施等方面问题，加大腾笼换鸟力度，承接核心区功能疏解，推动高精尖产业发展。抓好新首钢高端产业综合服务区建设，打造城市复兴新地标，带动西部地区转型发展。稳妥推进特色小城镇建设。加大对生态涵养区转移支付力度，完善多元化生态补偿机制，抓好长城国家公园体制试点，支持平谷、延庆创建国家森林城市，发展全域旅游，增强生态涵养功能和绿色发展能力。

　　集中力量抓好冬奥会、冬残奥会筹办工作。落实绿色、共享、开放、廉洁的办奥理念，全面推进各项筹备工作。加快国家速滑馆、国家高山滑雪中心、国家雪车雪橇中心等新建竞赛场馆建设，启动国家游泳中心等现有场馆改造，推进水利、电力、气象、通信、交通等配套基础设施建设。精心组织好平昌冬奥会、冬残奥会闭幕式接旗仪式和北京文艺表演，向世界发出北京邀请。征集北京冬奥会、冬残奥会吉祥物，启动官方赞助商征集和特许经营计划。加快冬季运动人才培养，提高冬季竞技体育水平。实施“共享冬奥”公众参与计划，推动冰雪运动进校园、进社区，推广普及群众冰雪运动。办好国际冬季运动(北京)博览会，打造京张体育文化旅游带，促进冰雪产业发展。

　　扎实推进2019年北京世园会筹办工作。完成园区场馆及配套设施建设和国内外招展任务，细化会期综合服务保障方案，带动旅游、园艺等相关产业发展。

　　认真做好对口支援合作工作。深入研究受援地区的资源禀赋、产业基础和脱贫需求，确保财政援助资金80%以上用于精准扶贫、精准脱贫，实施一批扶贫力度大、脱贫效应强的项目，支持和培育一批带动贫困户发展的产业合作组织和龙头企业，增强受援地区发展的内生动力。

　　(四)全力推进全国科技创新中心建设，加快构建高精尖经济结构。

　　科技创新是北京实现高质量发展的第一动力。要把科技创新和高精尖经济结构紧密结合起来，以国家战略需求引导科技创新方向，以科技创新引领高精尖产业发展，不断增强首都发展的创新力和竞争力。

　　央地协同推进重大科技任务。充分发挥北京推进科技创新中心建设办公室统筹协调作用，组织实施新一轮重点任务和项目。发布实施建设世界一流新型研发机构的政策举措，集聚全球创新人才及团队，建设好北京量子信息科学研究院、北京脑科学与类脑研究中心。推动国家重大科技基础设施布局建设和发展，承接“航空发动机”、“天地一体化信息网络”等重大项目。强化企业创新主体地位，在石墨烯、新能源汽车、智能电网等领域，新建一批国家技术创新中心、国家制造业创新中心。支持在京高校建设世界一流大学和一流学科，建设好高校“高精尖创新中心”等创新平台。支持基础科学中心建设，围绕前沿战略领域开展高水平原创研究。加快国家技术标准创新基地建设，增强质量技术创新能力。

　　高水平推进“三城一区”建设。编制完成“三城一区”规划，促进协同联动发展。中关村科学城要聚焦，以建设具有全球影响力的原始创新策源地和自主创新主阵地为目标，着力提高对全球创新资源的开放和聚集能力，着力补齐优质创新要素，着力促进不同创新群体深度融合，通过优化空间布局、打造创新型服务政府、强化城市创新形象，营造国际一流的创新创业生态，在建设全国科技创新中心中发挥带动引领作用。怀柔科学城要突破，建立国际化、开放式管理运行新机制，深化综合性国家科学中心建设，开工建设高能同步辐射光源等大科学装置，引导民间资本、社会力量、国际资源广泛参与，促进科研基础设施共建共享，构建从基础设施、基础研究、应用研究、成果转化到高精尖产业发展的创新链。未来科学城要搞活，鼓励入驻央企加大研发投入，引导企业建立有利于创新的公司治理结构，积极引入民营研发机构、创新企业、高校等多元主体，聚集提升投资孵化、科技服务等创新要素。北京经济技术开发区要优化提升，建立与三大科学城的对接转化机制，统筹大兴、通州等空间资源，与顺义、房山协同发展，抓好一批重大项目落地，提高外资引进和开放发展水平，打造创新型产业集群和中国制造2025示范区。

　　充分发挥中关村示范区引领支撑作用。加大先行先试力度，在科技成果转化、知识产权保护、科技金融改革、生物医药材料通关便利化等方面，争取实施新一批创新政策。落实以增加知识价值为导向的分配政策，增强科研人员的改革获得感。推进国家科技金融创新中心建设，完善创业投资政策，加大对小微企业融资支持力度，加强企业上市、挂牌培育工作。深化中关村人才管理改革，在朝阳望京、中关村大街、未来科学城和新首钢试点建设国际人才社区。完善创业服务体系，新增一批国家级双创示范基地，打造全过程的科技孵化服务链条。统筹“一区多园”发展，加强对分园创新发展的考核评价和产业定位引导，改革完善分园管理运行机制，发挥中关村发展集团在整合创新资源方面的市场化平台作用，探索引入国际化专业团队参与园区管理。

　　对标国际一流构建高精尖经济结构。落实新一代信息技术等10个高精尖产业发展指导意见，提升金融、文化创意、商务等现代服务业发展质量和水平。制定实施创新型企业培育壮大三年行动计划，支持行业领军企业、独角兽企业和隐形冠军企业发展壮大，做好重点企业“一对一”服务工作。推动军民融合深度发展，建设军民融合产业园和一批示范工程项目。设立产出效益、产业人口密度、研发投入强度、资源环境约束等产业指导标准，落实高精尖产业用地用房政策，实施鼓励企业加大研发投入、上市融资、产业投资、人才引进、外资开放等一揽子政策措施。通过统筹产业布局，推动全市产业向重点园区聚集、园区产业向主导产业聚集、主导产业向创新型企业聚集。

　　建立开放融合的科技创新机制。全面加强科技成果转化统筹协调与服务平台建设，与在京高校、科研院所建立深层次对接机制，做好科技成果在京落地承接服务工作。发挥科技创新基金引导作用，在科技领域积极推进政府和社会资本合作，鼓励多方资源更多投向基础研究和战略硬技术，促进科学家、企业家、投资人等深度合作。以开放共享为导向，支持高校与研发机构建设联合创新平台、协同创新研究院。扩大首都科技条件平台、创新券和开放实验室服务范围和领域，引导高校院所面向社会开放科技资源，为小微企业和创业团队提供研究开发、检验检测认证等科技服务。建设中国(北京)知识产权保护中心，深化与世界知识产权组织的合作，鼓励企业加快海外知识产权布局，参与国际标准研究和制定。积极参与国际大科学计划和大科学工程，增强北京在全球科技竞争中的影响力和话语权。

　　(五)加强城市精细化管理，下大气力治理“大城市病”。

　　贯彻习近平总书记提出的“城市管理要像绣花一样精细”的要求，以精治为手段，以共治为基础，以法治为保障，积极构建有效的超大城市治理体系。

　　提高城市精治共治法治水平。深化街道管理体制改革，建立职责清单，完善街道乡镇实体化综合执法平台，实现“街乡吹哨，部门报到”。完善街巷长制，发挥小巷管家作用，健全长效管理机制，深入推进以背街小巷为重点的精细化管理。充分运用信息化、智能化手段加强城市管理，实施北京大数据行动计划，建立城市大数据平台。推进智慧城市建设，开展副中心智慧城市试点。深化城市网格化管理，完善市级管理平台和市区街三级监督指挥体系。坚持社会共治，拓宽群众参与渠道，健全倾听群众意见工作机制，完善和整合各部门各单位服务热线，让公众、社会组织和企业在城市治理中发挥更大作用。完善城市管理领域法规、制度和标准。强化城管综合执法部门与各城市管理部门的工作衔接，完善与公安、消防、市场监管、安全生产等部门的联合执法机制，加快推进行政处罚权在规划国土、住房城乡建设部门内部集中行使。

　　坚决打好蓝天保卫战。以加强科学化、系统化、精细化、法治化管理为目标，以细颗粒物来源解析为基础，制定实施新一轮大气污染防治行动计划。完成环保垂直管理改革，加强基层执法力量，深入开展环保督察，进一步压实各级各部门责任。把高排放车治理作为重中之重，将低排放区由六环路内扩展到全市域，从严查处尾气排放超标车辆，严格落实高排放车和非道路移动机械禁行限行规定。有效管控裸地扬尘、道路扬尘、施工扬尘和建筑垃圾运输车辆，减少扬尘污染。实施环保技改工程和污染源达标排放行动计划，全面治理挥发性有机物污染。对重点企业严格排污许可证管理，实现“散乱污”企业动态清零。有序推进农村煤改清洁能源，基本实现平原地区“无煤化”。认真落实空气重污染应急预案，严格执行烟花爆竹安全管理规定，加强区域大气污染联防联控。传播生态文明理念、绿色生活知识，引导市民践行绿色生活方式。

　　坚持不懈抓好交通拥堵治理。继续实施缓解交通拥堵年度行动计划，努力改善出行条件。制定全市地面停车规划，实施公共区域停车管理网格化巡查，加强交通静态管理。开展学校医院周边等重点区域交通秩序整治，规范旅游等专线交通运营秩序，持续改善北京站、北京西站、北京南站交通转换环境。加强智能交通系统建设，扩大实施信号灯“绿波”工程，推进公共交通调度智能化。开展地下空间分层出让建设公共停车场试点，稳妥推进中心城区和城市副中心路侧停车电子收费。推动以轨道交通为主的公共交通体系建设，年内开通3段轨道新线，运营总里程达到630公里以上，市郊铁路里程达到238公里。实施轨道交通换乘通道改造工程，完善轨道交通和地面交通接驳换乘。新开和优化调整公交线路40条以上，新增公交专用道40公里以上。推进青年路北延等城市主干道建设，实施疏堵工程100项。完成900公里自行车道治理，加强共享单车规范管理，完善步道系统，中心城绿色出行比例提高到73%。

　　持续改善生态环境质量。实施新一轮百万亩造林绿化工程，新增造林绿化面积23万亩。推进节水型社会建设，启动水资源税改革试点，实施“两田一园”农业高效节水方案，对乡镇建立精细的水资源管理制度。珍惜用好南水北调水，促进地下水资源涵养和地下水位回升。深入实施河长制，落实污水治理和再生水利用第二个三年行动方案，完成非建成区84条段黑臭水体治理任务，全市污水处理率提高到93%，再生水年利用量达到10.7亿立方米。推进海绵城市建设。落实生活垃圾分类治理三年行动计划，扩大强制分类实施范围，示范片区覆盖率达到30%。探索实行差别化垃圾收费和补偿政策，促进垃圾减量。建成鲁家山餐厨垃圾处理厂、通州生活垃圾焚烧厂等设施，生活垃圾资源化率达到58%。建设土壤环境监测网络，完成全市农用地土壤污染详查，推进污染地块风险防控与治理。开展生态治理和修复，支持废弃矿山转型发展生态文化旅游业。

　　(六)进一步坚定文化自信，把全国文化中心建设好发展好。

　　文化是城市的灵魂，是人民美好生活的重要内容。要聚焦“一核一城三带两区”，有效保护、发掘、传承和利用北京丰富的历史文化资源，让文化深度融入城市发展，使北京成为文化底蕴厚重、艺术繁荣发展、社会风气高尚的城市。

　　推进首都文明建设。坚持以培育和践行社会主义核心价值观为统领，全面推进思想道德建设，旗帜鲜明地弘扬主旋律，倡导文明新风尚，亮出北京精气神。巩固和拓展“北京榜样”、“百姓宣讲”、“中国梦365个故事”等宣传品牌，深化文明城区、文明村镇、文明单位、文明家庭和文明校园创建工作，持之以恒倡导“光盘行动”，广泛开展环境、秩序、旅游、禁烟等公共文明引导行动，增强群众道德意识、文明意识、责任意识，提高城市文明水平。

　　保护城市历史文化风貌。贯彻习近平总书记“老城不能再拆了”的重要指示精神，研究制定推进老城整体保护实施方案，严格执行皇城保护规划，完善文物保护与周边环境管控的法规和机制，进一步明确各级政府、文物部门和各类主体的责任义务。统筹老城内房屋腾退和征收政策，配套建设定向安置房，做到在保护中改善民生，在改善民生中实现更好的保护。扎实推进中轴线申遗保护工作，抓好故宫周边重点文物腾退修缮。加强“三山五园”地区保护，实施北法海寺二期修缮工程，继续开展圆明园考古及展示。注重保护传统院落和胡同肌理，精心打磨每个历史文化街区。制定实施大运河文化带、长城文化带、西山永定河文化带保护建设规划，开工建设白浮泉、路县故城等遗址公园，开展箭扣长城二期等文物保护修复工程，启动琉璃河西周燕都遗址区搬迁腾退工作。推进香山双清别墅红色遗址群的保护、传承和利用。建立市级传统村落名录，加强规划保护，留住历史记忆、传统文化和乡土故事。支持老字号品牌传承发展。加强非物质文化遗产和历史典籍的保护利用，强化文物院落腾退后公益开放使用，鼓励相关单位、居民及公益机构参与保护利用，让文物活起来。

　　提升城市文化生活品质。推进公共文化服务体系示范区建设，继续实施文化惠民工程，深入落实“1+3”公共文化政策，完善公共图书、文化活动、公益演出等服务配送体系，举办2万场次市民系列文化活动。支持实体书店、书屋发展，开展全民阅读活动，营造书香京城。加强优秀近现代建筑和老旧厂房保护利用，为城市留有文化艺术空间。支持各类文艺院团做强做优，鼓励社会力量参与公共文化服务体系建设，丰富优质文化产品和服务供给，满足人民群众多样化、多层次的文化消费需求。

　　增强首都文化创造力影响力。深化文化体制改革，激发文化市场活力。推动文化与科技、金融、旅游、时尚、会展等相关产业融合发展，设立文化创新发展基金，积极培育新型文化业态，建设文化创意产业发展引领区。加大对重点作品、优秀原创作品的扶持奖励力度，推出一批体现中国精神、首都水准和北京特色的精品力作，勇于攀登文艺高峰。巩固做精北京国际设计周、国际电影节、国际音乐节和中国戏曲文化周等文化品牌。加强文化对外交流合作，办好“欢乐春节”、“北京之夜”等活动，推动文化走出去，展示首都文化魅力。

　　(七)持续保障和改善民生，切实增强人民群众获得感幸福感安全感。

　　围绕满足人民群众便利性、宜居性、多样性、公正性、安全性需求，坚守底线、突出重点、完善制度、引导预期，扎实办好重要民生实事，提高民生保障和服务水平。

　　努力办好人民满意的教育。坚持立德树人，加强师德师风建设，加大幼儿园、中小学师资培养力度，建设高素质专业化教师队伍。实施第三期学前教育行动计划，坚持公办民办并重，创新财政投入方式，大力发展普惠性幼儿园，年内新增学位3万个左右，积极应对学龄人口变化。加强学前教育管理，强化办园者主体责任，落实政府部门监管职责，实施幼儿园责任督学挂牌督导，引导家长参与管理，让孩子安全、家长放心。在重点新城和生态涵养区启动新建一批示范性学校，加大对学区制改革、集团化办学和乡村学校的支持力度，进一步缩小城乡、区域、校际差距。以中高考改革推进教育综合改革，减轻中小学生课外负担，推动教育理念、育人方式转变。坚持以人才培养为核心，推动高等教育内涵、特色、差异化发展。深化城教融合、校企合作，推动职业教育、继续教育、特殊教育和网络教育与首都经济社会发展需求更紧密结合，建设学习型城市。

　　全面推进健康北京建设。立足全人群和全生命周期两个着力点，提供公平可及、系统连续的健康服务。以电子病历和居民健康档案为主要内容，建设市区两级互联互通的全民健康信息平台。完善现代医院管理制度，开展公立医院薪酬制度改革试点。完善分级诊疗制度，每个区至少建设一个紧密型医联体，发展专科医联体，加强基层医疗卫生服务能力建设，做实家庭医生签约服务，完善预约挂号系统，严厉打击号贩子，让群众看病更方便。实施药品采购两票制和京津冀医用耗材联合采购，规范调整第二批医疗服务价格。推进以总额控制为主的多元复合式医保支付方式改革，在全市三级医院推广按病种分组付费。继续做好跨省异地就医住院费用直接结算服务。巩固推进国家卫生区创建工作，广泛开展全民健康行动和爱国卫生运动。加强重大传染病和新发突发传染病预防控制，开展慢性非传染性疾病筛查和干预服务。做好公民无偿献血工作。实施助产能力提升行动，提高母婴安全保障能力。传承弘扬中医药文化，推动名中医到基层社区开展服务。落实全民健身条例，建设群众身边的体育设施，扩大公共体育设施开放。扎实做好2019年男子篮球世界杯筹办工作。创建国家食品安全示范城市，全市80%以上的中小学校、托幼机构、养老机构食堂达到“阳光餐饮”标准。严厉打击各类食品药品违法行为，保障群众饮食用药安全。

　　完善租购并举的住房制度。严格执行各项房地产调控政策措施，加强市场监测和政策储备，促进房地产市场平稳运行。完成1200公顷住宅供地，扩大住房供给，稳定市场预期。发展住房租赁市场特别是长期租赁，推进集体建设用地建设租赁住房。鼓励产业园区建设职工集体宿舍，多渠道解决城市运行和服务保障行业务工人员住宿问题。规范住房租赁市场管理，落实承租人稳定居住和享受公共服务的各项保障政策。着力推进已供地290万平方米共有产权住房和600万平方米商品住房建设，尽快形成市场供应。建设筹集各类保障性住房5万套，完成棚户区改造2.36万户。

　　做好就业和社会保障工作。弘扬劳模精神和工匠精神，营造劳动光荣的社会风尚和精益求精的敬业风气，强化就业技能培训，不断提高劳动者素质。实施重点群体精准就业帮扶，做好相关企业职工分流安置，实现城镇新增就业36万人。实施全民参保计划，完善城乡居民基本医疗保险制度，健全城乡居民基础养老金和福利养老金标准动态增长机制，鼓励发展商业医保和企业年金，完善多层次社会保障体系。整合困难群众信息和相关救助资源，建立市区街三级困难群众帮扶救助机构。加强残疾预防和残疾人康复服务，持续推进无障碍设施建设，积极构建残疾人平等融合的社会环境。

　　提高养老服务能力。坚持养老服务事业与产业协同推进、居家养老与机构养老统筹发展，构建养老、孝老、敬老政策体系和社会环境。推进新建小区养老设施配置标准化，年内新建社区养老服务驿站150家，加强农村地区互助性养老服务设施建设。加大政府购买基本养老服务力度，建立综合性老年津贴、居家养老巡视探访等制度，推进养老护理师改革试点，扩大社会敬老优待内容。推进医养结合，深化政策性长期护理保险试点。持续提升养老服务质量，更好满足多元化养老需求。

　　加强和创新社会治理。实施基层社会治理规范化建设三年行动计划，完善城乡社区治理，健全自治、法治、德治相结合的乡村治理体系。实施社区基本公共服务“十大覆盖工程”，建设200个“社区之家”示范点，实现“一刻钟社区服务圈”覆盖90%以上的城市社区。开展社区服务社会化改革，提升社工队伍专业化职业化水平。加强社会心理服务体系建设，培养自尊自信、理性平和、积极向上的社会心态。推动志愿服务促进条例修订，促进志愿服务常态化制度化。坚持男女平等基本国策，维护妇女儿童、未成年人合法权益。贯彻落实党的民族宗教政策，加强各民族交往交流交融，积极引导宗教与社会主义社会相适应。支持国防和军队改革发展建设，继续深化国防动员和国防教育，推进优抚安置体制机制改革，不断巩固和发展首都军政军民团结。

　　深入开展平安北京建设。坚持总体国家安全观，以首善标准做好维护政治安全、反恐防恐、社会安全、公共安全、网络安全等各项工作，切实履行维护首都安全的重大职责。树立超大城市安全发展理念，弘扬生命至上、安全第一的思想，加强安全宣传教育，提高防灾减灾救灾能力。严格落实安全生产责任制，以“三合一”、“多合一”场所为重点，深入开展城市安全隐患治理三年行动计划，强化消防安全、建筑施工、道路交通等重点领域整治，坚决遏制重特大安全事故。保障水电气热等城市生命线安全运行。强化社会矛盾风险预测预警预防，完善人民调解、行政调解、司法调解衔接联动机制，提高信访工作专业化、法治化、信息化水平，依法及时就地解决群众合理诉求。巩固深化立体化、信息化社会治安防控体系，依法打击和惩治黄赌毒黑拐骗等违法犯罪活动，组织开展扫黑除恶专项斗争，全力确保首都安全稳定。

　　(八)大力推进改革开放，构筑体制机制新优势。

　　以纪念改革开放40周年为契机，总结经验，乘势而上，围绕创造优良营商环境、提供优质公共服务精确发力，切实推动重大改革任务举措落地，全面提高开放发展能力和水平。

　　以营商环境改善激发市场主体活力。落实改革优化营商环境实施方案，对各区实行营商环境评价。深化“放管服”改革，做好国务院取消、下放行政审批事项的落实衔接工作。强化企业投资主体地位，实现九成以上企业投资项目、全部备案项目审批权限下放到各区，备案制投资项目取消所有前置条件。持续推进“多证合一”，探索开展“证照分离”改革，扩大全程电子化登记试点范围。强化事中事后监管，完善社会信用体系，加大守信激励和失信惩戒力度。深化金融改革，支持多层次资本市场创新，增强服务实体经济能力。优化金融业空间布局，推动金融街与丽泽金融商务区一体化发展。加强政府债务管理，履行地方金融监管职责，坚决打击违法违规金融活动，守住不发生系统性金融风险的底线。完善国有资产管理体制，做好国有资本投资和运营公司试点，加快国有经济布局调整和战略重组，推动国有资本做强做优做大。大力压缩国有企业管理层级，发展混合所有制经济，推动一级企业整体上市或主业上市。下大气力激活民间投资，着力破除歧视性限制和各种隐性障碍，支持民营企业参与政府与社会资本合作项目，引导社会资本进入医疗、教育、养老、体育等领域。着力降低实体经济成本，继续清理涉企收费。落实税收优惠政策，优化纳税服务。加强产权保护，激发和弘扬企业家精神，构建亲清新型政商关系。

　　以关键环节改革提高公共服务能力。聚焦群众关心的办事不方便问题，推动政务服务更透明、更便捷。完善四级政务服务体系，规范事项清单，推进办事指南标准化、场景化，完善政务公开惠民便民地图。推动公共服务事项进驻各级政务服务中心，变“集中审批”为“集成服务”。完善网上政务服务大厅功能，推进跨层级、跨部门信息互认共享，提升网络终端、移动终端和自助服务终端办事服务能力，解决“最后一公里”问题，让信息多跑路、群众和企业少跑腿，办事像“网购”一样方便。继续深入清理“奇葩证明”。全面加强绩效管理，建立以质量和成本为重要考核内容、以结果导向配置公共资源的绩效管理机制，提高财政资金使用效益。稳步实施市区财政事权与支出责任划分改革，有效提高基本公共服务能力。

　　以更高标准推动全面开放。实行更加积极主动的开放战略，制定国际化服务环境和服务能力提升计划，营造法治化、国际化、便利化的发展环境。加强国际交往中心重要设施和能力建设，全力做好国家重大主场外交活动服务保障工作。全面完成服务业扩大开放两轮试点任务，抓好试点示范区和示范项目建设，培育形成一批新模式新业态，打造服务业“北京品牌”。全面提升首都金融业对外开放能力，支持国际金融机构在京新设外商独资或合资金融机构。完善144小时过境免签配套政策，扩大旅游消费。积极为港澳台同胞和外籍人员就学就医提供便利，深化外事侨务引资引智工作。持续推进服务贸易竞争力提升工程，办好第五届京交会。完善天竺综保区功能，高水平规划建设北京新机场综保区，提升贸易便利化水平。制定北京“一带一路”三年行动计划，加强双向投资促进平台建设，与沿线国家联合建设科技创新园区，拓展开放的广度和深度。

　　三、全面加强政府自身建设

　　五年来，政府自身改革和建设取得重要进展。扎实开展党的群众路线教育实践活动、“三严三实”专题教育、“两学一做”学习教育，干部队伍理想信念更加坚定，纪律规矩意识明显增强。共办理市人大代表议案17项、建议5591件，办理市政协提案4926件，提请市人大常委会审议地方性法规18项，制定修改废止政府规章60项。严格落实中央八项规定精神，大力整治“为官不为”、“为官乱为”问题，政府系统廉政建设和反腐败斗争不断深入。

　　各位代表！新时代要有新气象、新作为，必须有新视野、新担当。要全面深入学习贯彻党的十九大精神，坚持刀刃向内，敢于自我革命，加快转变政府职能，努力建设法治政府、创新政府、廉洁政府和服务型政府，进一步提高政府公信力和执行力，让中央放心、让人民满意。

　　提高政治站位。自觉以习近平新时代中国特色社会主义思想为指引，牢固树立“四个意识”，坚定“四个自信”，坚决维护习近平总书记在党中央、在全党的核心地位，坚决维护党中央权威和集中统一领导，始终在政治立场、政治方向、政治原则、政治道路上同以习近平同志为核心的党中央保持高度一致。深入开展“不忘初心、牢记使命”主题教育，坚定理想信念，更加自觉地践行党的根本宗旨。深入思考新时代的首都职责使命，着眼中华民族伟大复兴大背景、京津冀协同发展大格局谋划和推动首都工作，自觉在服务国家大局中提高自身发展水平。

　　加强依法行政。要把政府工作全面纳入法治轨道，不断提高运用法治思维和法治方式推动工作的能力。认真执行市人大及其常委会的决议决定，自觉接受人大和政协监督。健全行政决策机制，对涉及重大公共利益、市民切身利益的事项，要广泛听取各方面意见。推动非机动车管理、科技成果转化、安全生产等重点领域立法。严格规范文明执法，全面落实行政执法责任制。加大政府信息公开力度，加强政策解读，强化舆情分析应对，主动回应社会关切，接受人民监督。加强法治教育，完善公共法律服务体系，让尊法学法守法用法成为全社会的共同追求和自觉行动。

　　增强工作本领。推动首都高质量发展，迫切需要一支高素质专业化的干部队伍。强化质量意识，建立质量导向的工作机制，切实把高质量要求体现到政府工作的方方面面。提高创新能力，不断掌握新知识、熟悉新领域、开拓新视野，善于运用大数据、物联网等新技术推动政府管理创新。提高专业能力，培育专业精神，增强对产业、技术、管理的敏感性，更多用专业力量、专业方法解决发展中的问题。提高开放能力，对标国际一流标准，关注世界发展前沿，积极利用国际国内两个市场、两种资源推动发展。提高工作统筹能力，坚持稳中求进，强化底线思维，增强忧患意识，防范各种风险挑战，牢牢把握工作主动权。

　　持续改进作风。严格执行中央八项规定及其实施细则精神和市委贯彻落实办法，坚决反对特权、不搞特权，坚决防止“四风”反弹回潮，坚决纠正形式主义、官僚主义顽症。发扬唯实求真精神，大兴调查研究之风，扑下身子、沉到一线，精选主题蹲点调研，既要“身入”更要“心至”，察实情、体民忧、解难题，提高决策的精准度和有效性。走好新形势下的群众路线，更加善于做群众工作，学会与群众面对面、心贴心，始终与人民群众站在一起、想在一起、干在一起，努力赢得群众的信赖和支持。健全“督考合一”工作机制，把群众满意度作为政府绩效考评的重要依据，加大群众评议和第三方评估力度。雷厉风行抓落实，功成不必在我，脚踏实地、久久为功，以钉钉子精神确保每一项工作、每一条措施都落到实处。

　　建设廉洁政府。落实全面从严治党主体责任，推动政府系统党风廉政建设向纵深发展。深化廉政风险防控，做好巡视发现问题的整改。持续开展专项整治，以深化改革铲除滋生腐败的土壤。严防严惩重点领域腐败问题，加强公共资金、国有资产、国有资源和领导干部经济责任审计监督，加大对权力运行的规范和制约。严格执行廉洁自律准则和纪律处分条例，强化监督执纪问责，保证干部清正、政府清廉、政治清明。

　　各位代表，新时代蓝图已绘就，新征程奋进正当时。让我们更加紧密团结在以习近平同志为核心的党中央周围，在中共北京市委的坚强领导下，紧紧依靠全市人民，不忘初心、牢记使命，锐意进取、埋头苦干，加快建设国际一流的和谐宜居之都，奋力谱写实现“两个一百年”奋斗目标和中华民族伟大复兴中国梦的北京篇章！
